# Ligation of the canine mesovarium with a resorbable self-locking device: a multicentre study

**DOI:** 10.1186/s13028-026-00884-3

**Published:** 2026-09-19

**Authors:** Livia de Paula Coelho, Matheus Roberto da Mota Costa, Renato Moran Ramos, Andre Lacerda de Abreu Oliveira, Bruno Watanabe Minto, Guilherme Galhardo Franco, Julianna Thuroczy, Rogério Luizari Guedes, Peterson Triches Dornbusch, Odd Viking Höglund

**Affiliations:** 1 Faculdade de ciências agrárias e veterinárias, UNESP – Jaboticabal/SP, Rua Minas Gerais, 1223, setor Samuel Graham, Jataí, GO Brazil; 2 Universidade Iguaçu – UNIG campus V, Itaperuna/RJ, BR 356, KM 02, Itaperuna, RJ 28300-000 Brazil; 3 UENF, Universidade Estadual do Norte Fluminense Darcy Ribeiro, Av. Alberto Lamego, 875 - Parque California, Campos dos Goytacazes, RJ Brazil; 4 Faculdade de ciências agrárias e veterinárias, UNESP – Jaboticabal/SP, Via de acesso prof. Paulo Donato Castellane, Jaboticabal, SP Brazil; 5 Universidade Federal do Espírito Santo (UFES), Rua Dulcino Pinheiro 45, centro, Alegre, ES Brazil; 6 Animal Health Center Budafok, Kossuth Lajos u. 52, Budapest, H-1221 Hungary; 7 Veterinary School at Federal University of Paraná (UFPR), Rua dos Funcionários 1540, Setor de Ciências Agrárias, Curitiba, Paraná Brazil; 8 Department of Clinical Sciences, SLU, Swedish University of Agricultural Sciences, UPPSALA, Box 7054, 750 07 Uppsala, Sweden

**Keywords:** Bitch, Dog, Oophorectomy, Ovariectomy, Ovariohysterectomy, Spay, Surgical haemostasis

## Abstract

**Background:**

Haemorrhage from the ovarian pedicle is a known complication at removal of ovaries in dogs. A resorbable self-locking device designed for surgical ligation was previously developed, aimed to keep the advantage of a self-locking loop at ligation and avoid the chronic tissue reactions of non-resorbable materials. The primary aim of the study was to continue to evaluate the feasibility and clinical performance of the device for ligation of the canine mesovarium in a prospective multicentre controlled study. Secondary aims were to compare perioperative complications, ligation time, and duration of surgery between the device and a traditional double ligature technique.

**Results:**

In total, 72 intact female dogs destined for elective ovariohysterectomy were included in the study. The device was tested in 36 dogs across four centres, and 36 dogs were used as controls. Across all centres, body weights of device and control groups were 14.5 ± 1.0 kg and 14.9 ± 1.3 kg, respectively. The device group was 2.5 ± 0.3 years old and controls were 3.6 ± 0.4 years old. Estimating mean times from the linear mixed models gave ligation times for device and control groups of 4.4 ± 1.3 minutes and 4.5 ± 0.4 minutes, respectively. Duration of surgery for device and control groups were 21.3 ± 4.0 minutes and 16.7 ± 0.7 minutes, respectively. Both surgery and ligation times showed a significant centre-specific treatment effect. Two cases of intraoperative bleeding from the ovarian pedicles were observed in the control group. No postoperative complications were observed and all dogs recovered uneventfully.

**Conclusions:**

The device could be used for compression of tissue of the ovarian pedicle, provided haemostasis and adequate support of tissue throughout the healing period. Time for double ligation and duration of surgery did not differ between groups. Centres with previous experience of using the device recorded shorter ligation times and duration of surgery, which suggests a learning curve.

**Supplementary Information:**

The online version contains supplementary material available at https://doi.org/10.1186/s13028-026-00884-3.

## Background

One recognised intra- or postoperative complication of ovariectomy in dogs is haemorrhage from the ovarian pedicle [1]. This occurs more often in large and deep-chested breeds [2] and the duration of surgery is longer in larger dogs [3, 4]. Ligation of the ovarian pedicle and removal of ovaries is a part of the surgery with intense noxious stimuli, which causes a surgical stress response, associated to postoperative pain [5–9]. The perceived challenge of the procedure was described in a survey of final year veterinary students where 81% indicated they were most worried about canine ovariohysterectomy [10]. Several methods to provide haemostasis exist, ranging from traditional ligation to electrosurgery, vessel sealing devices, and new and improved techniques are being developed.

A cable tie or tie wrap, a self-locking loop, is a flexible band with a locking case at one end. Cable ties became popular in veterinary surgery in the 1970s. It has been suggested that duration of a bitch spay surgery can be reduced if self-locking loops are used to ligate the mesovarium [11–13]. However, traditional cable ties are not designed for surgery, are made without declared good manufacturing practice (GMP) and can contain substances not suited for long-term implantation. The material is nylon, a non-resorbable material that may cause chronic tissue reactions [14–18].

A resorbable self-locking device designed for surgical ligation was developed in a university research project, aimed to keep the advantage of a self-locking loop at ligation and avoid the chronic tissue reactions of non-resorbable materials. It was first manufactured in the resorbable polymer polydioxanone [19–21] and later in a resorbable block co-polymer of glycolide (GA) and trimethylene carbonate (TMC) [22–24] equivalent to the resorbable suture Maxon™ [25]. A previous feasibility study at one centre of 21 dogs suggested the device can be used to ligate the canine mesovarium. Compared to traditional suture ligation with one encircling ligation, the use of the device enabled a reduced time to ligate the ovarian pedicle and duration of surgery [4]. In addition to ligation at canine gonadectomy procedures, the device has also been subjected to pilot tests of cholecystectomy [26], lung lobectomy and lung biopsies [27–30] and equine laparoscopic cryptorchidectomy [31].

Previous studies have demonstrated feasibility and potential efficiency advantages of the device in single-centre settings with limited numbers and controlled conditions, but it remains unclear whether these findings are reproducible across different clinical environments, surgeons and practice settings. We hypothesised that the device would provide effective haemostasis and demonstrate comparable surgical performance to traditional ligation techniques when used across multiple centres. The primary aim of the study was to continue to evaluate the feasibility and clinical performance of the device for ligation of the canine mesovarium in a multicentre setting. Secondary aims were to compare perioperative complications, ligation time, and duration of surgery between the device and a traditional double ligature technique.

## Methods

### Ethical permits

An ethical permit was granted for each centre: Dr da Mota Costa & Dr de Abreu Oliveira, Campos dos Goytacazes, Rio de Janeiro, Brazil (UENF-CEUA 435813); Dr. J. Thuróczy, Hungary (PEI/001/652-2/2015); Dr. Minto, São Paulo, Brazil (012196/17); Dr R. Guedes & Dr. P. Dornbusch, Curitiba, Paraná, Brazil (CEUA Agrárias – UFPR 47/2016).

### Animals

Inclusion and exclusion criteria: Privately owned dogs or “Spay and adopt”, female dogs destined for gonadectomy, were included in this prospective study. If privately owned dogs were used, an owner’s consent was signed. Exclusion criterion was if the owner declined to participate in the study. There were no limits regarding age or bodyweight of dogs.

Each centre included about the same number of dogs tested with the device and controls. Each study centre organized the schedule of the surgical procedures for each group in an alternating allocation sequence, and the dogs for each group were selected based on the order of completion of the preoperative evaluation and the scheduled date of the procedure.

### Anaesthesia

Dogs were premedicated and anaesthesia was induced and maintained according to each centre’s preferences.

Centre A: Acepromazine (IM, 0.08 mg/kg), meloxicam (SC, 0.2 mg/kg), pre-operative morphine (IM, 0.5 mg/kg), epidural (lidocaine, 5 mg/kg).

Centre B: Combination of Ketamine hydrochloride at 5 mg/kg – Diazepam 0.1 mg/kg – Medetomidine 20 µg/kg IV.

Centre C: Acepromazine (IM, 0.08 mg/kg), meloxicam (SC, 0.2 mg/kg), pre-operative morphine (IM 0.5 mg/kg), epidural (lidocaine, 5 mg/kg).

Centre D: morphine sulphate (0.5 mg/kg, IM). During surgery, a continuous infusion of FLK (fentanyl citrate at 10 µg/kg/min, lidocaine hydrochloride at 50 mg/kg/min, ketamine hydrochloride at 10 µg/kg/min) was given. In the immediate postoperative period, meloxicam (0.2 mg/kg) and tramadol hydrochloride (2.0 mg/kg) was given SC.

A heat source was used if the body temperature was below normal in the perioperative period. Postoperative analgesia (meloxicam) was prescribed for 2–5 days (centres A – D); in addition, centre D prescribed tramadol hydrochloride (2.0 mg/kg, every 8 h, 3 days).

### The resorbable device

**Fig. 1:**
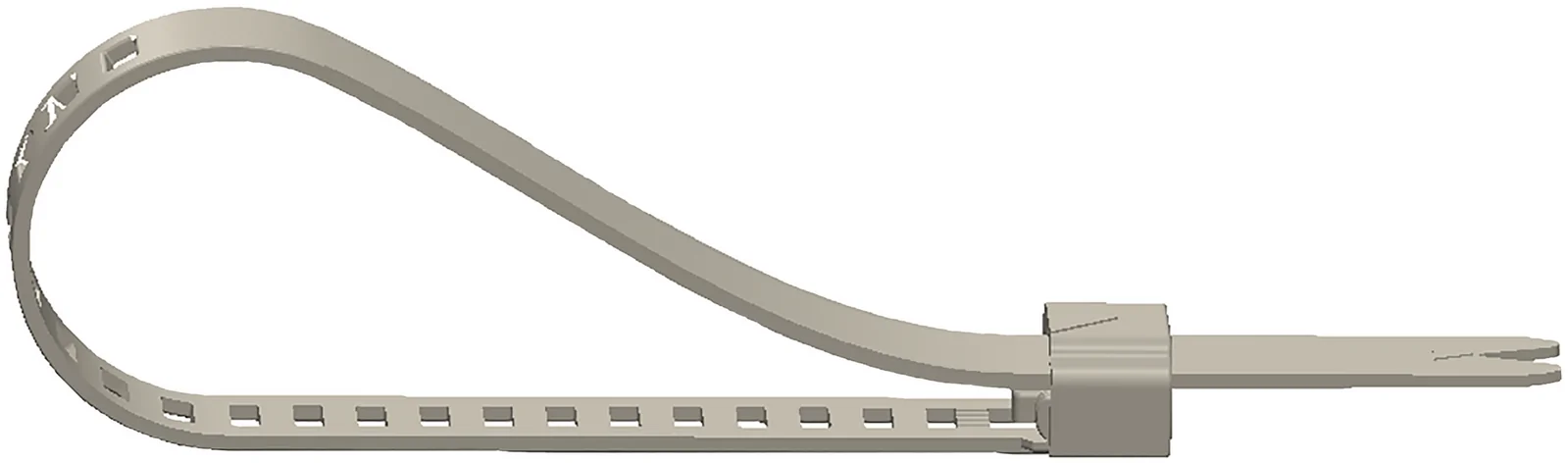
Illustration of the resorbable device. This consisted of a flexible band, in part perforated, and a case with a locking mechanism where the band could be introduced and pulled through

The design of the device has been reported previously. The device (LigaTie^®^, legal manufacturer Resorbable Devices AB, Uppsala, Sweden, www.LigaTie.com) consisted of a flexible band, partially perforated along its length, and a locking case (Fig. 1). The band was passed through the case to form a loop around the tissue. When the locking mechanism engages with the perforations, the band can only be advanced in one direction, allowing progressive tightening of the loop. Design features for tissue engaging properties, aimed to achieve a secured tissue grip, were added to the locking case to increase friction between device and tissue that was compressed inside the loop of device.

The resorbable polymer, a block co‑polymer of glycolide (GA) and trimethylene carbonate (TMC), was injection-moulded into device components. The devices were packaged in pairs in Tyvek pouches under clean-room conditions and sealed in aluminium foil pouches after vacuum drying. Sterilisation was carried out using electron beam radiation at a dose of 25 kGy.

### Surgery

At each centre, ovariohysterectomies were done by an experienced surgeon. Dogs were placed in dorsal recumbency in the Trendelenburg position and an incision was made along the midline, caudal to the umbilicus. Uterine horns and ovaries were located and exposed manually. For ligation of the ovarian pedicles, the self-locking device LigaTie^®^ or traditional resorbable suture (i.e. polyglactin 910 or polyglycolic acid) was used.

#### Device group

A hole was made in the broad ligament, at the junction of the mesovarium/mesometrium, the flexible band was applied around the mesovarium (ovarian pedicle) and the loop was tightened. For improved grip when tightening the loop, a needle holder could be applied to the flexible band. After tightening the loop of the device, haemostatic forceps were applied in-between the device and the ovary, and the ovarian pedicle was transected distal to the implanted device, close to the forceps. The stump was inspected for haemostasis. If the mesovarium was bleeding, instructions were to tighten the loop further, instead of applying a new device. No haemostatic forceps were applied to crush tissue of the ovarian pedicle before ligation with the device.

The procedure was repeated on the contralateral side removing the second ovary, following the same steps as previously described. Then haemostasis was verified a second time in the first ovarian pedicle, and the excess band protruding from the locking case was cut off on both sides. A short segment of the flexible band remained at the locking case to allow grasping the flexible band with a needle holder and enable further tightening of the loop, if needed.

The mesometrium was ligated if deemed necessary with resorbable suture material of the surgeon’s choice. An additional ligation of the uterine arteries, cranial to cervix, in addition to the circular ligation, was added with resorbable suture material of the surgeon’s choice. Tissue was transected and haemostasis was verified.

#### Control group

**Fig. 2:**
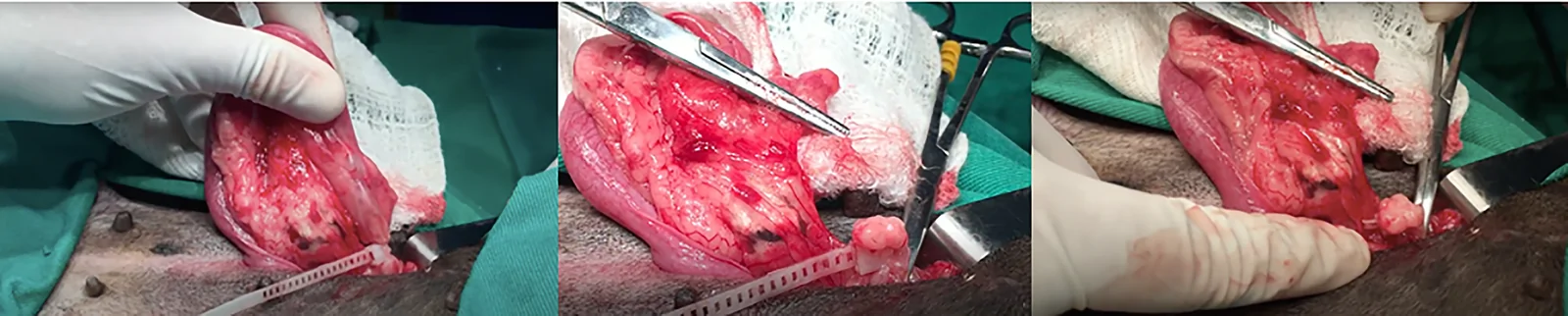
The resorbable self-locking loop was placed around the ovarian pedicle and tightened. Tissue was transected, and after haemostasis was verified, the excess band was cut off. Forceps were attached to edge of mesovarium to facilitate inspection of haemostasis

Tissue of the ovarian pedicle was double ligated with resorbable suture. First, the mesovarium was crushed with a haemostatic forceps and a groove was made. A ligature was placed in the groove of the mesovarium and a circular ligature was applied. An additional anchored ligature, figure-of-eight, was placed distal to the first circular ligature. Forceps were applied in-between the distal ligature and the ovary; thereafter the ovarian pedicle was transected distal to the ligatures, close to the forceps. The tissue was inspected to verify haemostasis. If intraoperative (or postoperative) bleeding was identified, an additional ligature was added (Figure 2).

### Definition of times

Duration of surgery was defined as from start of skin incision to last skin suture.

In the device group, the definition of time for ligation was from identification of the uterine horn (start) until transection of the second ovarian pedicle, verification of haemostasis and cutting of the excess band (stop).

In the control group, the definition of time for ligation was from identification of the uterine horn (start) until transection of tissue and verification of haemostasis of the second ovarian pedicle (stop).

Perioperative follow-up was daily control clinical examination (measure rectal temperature, perform abdominal palpation and visual examination of the surgical wound for dehiscence, bleeding or signs of infection) on minimum days 1, 2 and 10, at removal of sutures. Outcome measures were complications in the form of intraoperative or postoperative bleeding, ligation time and duration of surgery.

### Statistics

Descriptive statistics for age and bodyweight of dogs were calculated for each centre and for the pooled treatment groups and were reported as mean ± standard error.

Surgery and ligation times were log-transformed and modelled with a linear mixed model. The treatment factor, dog weight and dog age were included as fixed factors. The centre was included as a random factor with a random intercept and a random centre-specific treatment effect. The fixed factors were tested with a Type II Wald F-test with Kenward-Roger degrees of freedom and estimated marginal means per treatment were estimated and compared. Model assumptions were tested with a Shapiro-Wilk test for normality of residual terms and visual inspection of diagnostics graphs for constant variances. A centre-specific treatment effect was tested with a likelihood-ratio test by comparing a mixed model with a centre-specific treatment effect to a mixed model with only a random intercept. Analysis was done in R version 4.5.3 and RStudio 2026.04.0 + 526, with the R packages tidyverse, car, emmeans, and lme4. Data were reported as means ± SE.

## Results

In total, 72 privately owned or “spay and adopt” intact females destined for elective OHE and adoption were included in the study (Table 1).

**Table 1:** – Body weights and age of the 72 dogs at four centres, control or device group. Centre C had two cases of uterine pathology (pyometra), one dog in each group (control/device)

Centre	Number of dogs control / device	Bodyweight control dogs, kg	Bodyweight device dogs, kg	Age control dogs, years	Age device dogs, years
A	10 / 10	14.1 ± 1.9	12.2 ± 1.6	4.6 ± 0.5	2.6 ± 0.7
B	13 / 12	19.1 ± 2.8	19.9 ± 1.7	3.5 ± 0.7	2.2 ± 0.4
C	10 / 10	11.5 ± 1.4	12.7 ± 0.8	2.6 ± 0.7	2.5 ± 0.6
D	3 / 4	10.3 ± 2.9	8.1 ± 1.3	4.3 ± 0.9	2.8 ± 1.2
Total	36 / 36	14.9 ± 1.3	14.5 ± 1.0	3.6 ± 0.4	2.5 ± 0.3

Body weights of device and control groups were 14.5 ± 1.0 kg and 14.9 ± 1.3 kg, respectively. The device group was 2.5 ± 0.3 years old and controls were 3.6 ± 0.4 years old.

Estimating mean times from the linear mixed models gave that ligation times for device and control groups were 4.4 ± 1.3 min and 4.5 ± 0.4 min, respectively.

Duration of surgery for device and control groups were 21.3 ± 4.0 min and 16.7 ± 0.7 min, respectively. Both surgery and ligation times showed a significant centre-specific treatment effect (Additional files 1–4: Statistical model results (pdf), Boxplot centres and time (pdf), sheet testing centre effect: models-results-ligation-time-used2.xlsx, models-results-surgery-time-used2.xlsx). There was no significant treatment effect in the tested model.

Staff at centres A & C had previous experience of using the device. Centre C had two cases of uterine pathology (pyometra), one dog in each group (control/device).

Two cases of intraoperative bleeding from the ovarian pedicles were observed in the control group (Table 2). One was from the right ovarian pedicle (Centre A, six years old, bodyweight of 24 kg) and one from the left ovarian pedicle (Centre C, three years old, bodyweight of 14 kg). The tissue was re-ligated, haemostasis was confirmed and the remaining surgical procedure was uneventful. No postoperative complications were observed and all dogs recovered uneventfully.

**Table 2:** – Results of the canine ovariohysterectomies involving device (*n* = 36) and controls (*n* = 36). Time for ligation of the ovarian pedicles, duration of surgery and reported intraoperative bleeding from the ovarian pedicle. Time in minutes

Centre	Ligation controls	Ligation device	Surgery controls	Surgery device	Intraoperative bleeding controls/ device
A	4.1 ± 0.8	2.9 ± 0.3	17.8 ± 1.5	17.9 ± 1.8	1 (ROP) / 0
B	5.9 ± 0.4	8.3 ± 0.7	17.9 ± 0.8	30.5 ± 1.3	0 / 0
C	3.7 ± 0.2	2.4 ± 0.1	15.7 ± 1.4	13.6 ± 0.4	1 (LOP) / 0
D	4.3 ± 1.3	7.2 ± 1.3	18.3 ± 2.3	27.5 ± 1.0	0 / 0
Total	4.5 ± 0.4	4.4 ± 1.3	16.7 ± 0.7	21.3 ± 4.0	2/0

Centre A & C had previous experience of using the device. Pyometra: At centre C there were two cases of pyometra, one in each group. ROP & LOP: Right and left ovarian pedicle, respectively

## Discussion

The results of this multicentre test of the resorbable self-locking loop LigaTie^®^ showed the device could be used for compression of tissue of the ovarian pedicle, provide haemostasis and adequate support of tissue throughout the healing period.

The risk of haemorrhage from the ovarian pedicle is a known complication at ovary removal [1, 2] and the procedure was therefore deemed suitable for this test of the resorbable ligation device. In the present multicentre study, the device compressed tissue and provided haemostasis as intended during healing of tissue. The results were in agreement with previous studies where the device was tested for canine gonadectomies [4, 21–23] as well as equine laparoscopic cryptorchidectomy [31]. The results of the study confirmed that sufficient mechanical strength of the device was retained during healing time of the blood vessels [24] and allowed adequate time beyond proliferative phases of wound healing of the ligated tissue [32].

No intra- or postoperative bleeding was observed in the device group. In the control group, two cases of intraoperative bleedings were observed and treated uneventfully. This agrees with previous reports of intraoperative complications at female gonadectomies in canines. In one study, where the surgery was performed by clinicians in a non-teaching institution, intraoperative bleeding from the ovarian pedicle occurred in 10% of dogs [33]. In studies of neutering in female dogs where the surgery was performed by students, major intraoperative haemorrhage of the ovarian pedicle was reported in 4% to 22% of the cases [2, 3, 10, 34]. The two control dogs where intraoperative bleeding was observed could be described as medium and large, which was in agreement with previous studies where risk of complications were considered high with large breed dogs. Large dogs are recognised as more challenging because of their deeper chests with a larger distance from skin incision to the tissue to ligate and transect [2, 4].

The use of haemostatic forceps on tissue to be ligated and transected differed between groups. In the device group, tissue was not crushed before application of the device, because this could contribute to haemostasis independently of the device and thereby confound assessment of its effect. In contrast, in the control group, the ovarian pedicle was clamped prior to ligation to create a groove for suture placement. In addition, minor differences in surgical technique were present, such as the timing of transection and trimming of suture or device material. However, these differences are inherent to the respective techniques and are unlikely to have substantially influenced the primary outcomes. Overall, the findings show that the device was able to achieve adequate haemostasis of the ovarian pedicle under the conditions of this study.

Standard self-locking loops for electrical cables (cable ties) are sometimes used by practitioners in surgery but there is a risk of chronic tissue reactions [14–18]. Therefore the use of traditional cable ties in surgery is strongly discouraged [35], as is the use of non-resorbable material for ligation purposes [36–40]. The tested device was made of resorbable polymers. Establishing the safety of a surgical device requires that a number of devices are tested in vivo, and an important aspect of biocompatibility is the success of an implantable medical device in fulfilling its intended function [41, 42]. Canine gonadectomy is an elective surgical procedure that has a high level of standardisation and was considered suitable for tests of the ligation device.

It may be challenging to standardise groups in a multicentre study. Although an alternating allocation order was used, age of animals varied between groups. Brazil, where surgeries at three centres were performed, has an estimated 30 million abandoned animals. All three centres, despite being located in different regions, have permanent population control programs for abandoned animals. The elective surgical procedure of canine gonadectomy in “spay-and-adopt” settings offered an opportunity to reduce the use of experimental animals, which is important for ethical reasons and in agreement with 3R [43].

There is a reduced risk of complications during canine OHE with increased experience. One study showed a significant reduction in the number of complications encountered by veterinarians between 6 and 12 months after completing their education [44]. In the present study, only experienced surgeons performed the surgical procedures and ligation in the control group was performed with traditional suture techniques. At summation of groups across centres, duration of surgery and ligation time between device and control groups did not differ.

A statistical comparison between centres was outside the intended aims of the study. However, centre A and C had previous experience of the device, whereas surgeons of centre B and D were first-time users of the device. This difference suggests procedures may have varied across centres. Additional statistical analysis suggested there was indeed a centre-specific treatment effect (additional files 1–4).

This centre-specific treatment effect suggested there was a learning curve, namely that the device enabled a shortened duration of surgery if the user had experience of the technology. The observed centre-specific treatment effect may reflect differences in prior experience with the device, although this was not formally investigated. This result should be interpreted cautiously because the study material was limited and did not aim to determine whether if previous experience of using the device affected outcome. Our findings suggest the device can be used safely for ligation, even when surgeons have not been trained to use the device. The basic surgical setting was familiar for all surgeons because canine OHE is one of the most common surgical procedures that veterinarians perform. To determine whether the device, when used by novice surgeons, affects ligation time and duration of surgery remains to be studied.

One aim to advance new surgical techniques is to reduce duration of surgery and it is also essential to minimize risk of complications. In a study by Schwarzkopf, the time for electrocoagulation vs. a double ligation of one ovarian pedicle was 2 min and 22 s and 4 min and 10 s, respectively [45]. In a previous study where the resorbable self-locking device was used, time for ligation of both ovarian pedicles was about 3.5 min [4]. In the present study, ligation time was 4.7 to 5 min, in agreement with Schwarzkopf’s electrocoagulation results, i.e. doubled, corresponding to both ovarian pedicles. In the present study, ovarian pedicles were double ligated in control dogs, which differed compared to the previous study where single ligation was used [4]. However, comparison with other studies should be done cautiously because multiple factors may add to variability, such as definition of times, ligation methods, single or multiple surgeons and centres.

Our study had limitations: inclusion criteria were broad, anaesthetic and perioperative analgesic protocols varied between centres, use of haemostatic forceps varied between groups, and the number of cases were limited and not evenly distributed between centres. The low number of bleeding events limits statistical power, and results should be interpreted with caution. There was no significant treatment effect in the mixed-effects model. However, the small number of centres may have limited statistical power to detect a centre-specific treatment effect. No power analysis was performed, and age of dogs was not matched between groups, device versus controls. The study was prospective, but no attempt was done to evaluate outcome of dogs in a blinded fashion. At centre C two of the bitches (one from each group) had an enlarged uterus. However, the inclusion of the two pyometra cases did not affect the main conclusion of the study.

## Conclusions

The device could be used for compression of tissue of the ovarian pedicle, provide haemostasis and adequate support of tissue throughout the healing period, which was in agreement with previous pilot tests. Time for double ligation and duration of surgery did not differ between groups. Centres with previous experience of using the device recorded shorter ligation times and duration of surgery, which suggests a learning curve. 

## Supplementary Information

Below is the link to the electronic supplementary material.

Supplementary Material 1: Additional file 1: Statistical model results (pdf)

Supplementary Material 2: Additional file 2: Boxplot centres and time (pdf)

Supplementary Material 3: Additional file 3: Statistical models ligation times (models-results-ligation-time-used2.xlsx)

Supplementary Material 4: Additional file 4: Statistical models surgery times (models-results-surgery-time-used2.xlsx)

## Data Availability

The datasets used in the current study are available from the corresponding author on reasonable request.

## Declarations

### Prior publication

Data have not been published previously.

## Abbreviations

LOP: Left ovarian pedicle
ROP: Right ovarian pedicle
