# Supplementary material for: Ligation of the canine mesovarium with a resorbable self-locking device: a multicentre study
Source: Acta Vet Scand. 2026 Sep 19;68:41. doi: 10.1186/s13028-026-00884-3 (PMC13613791; doi:10.1186/s13028-026-00884-3)
Supplement: Supplementary file 1 [file 13028_2026_884_MOESM1_ESM.pdf]

Supplementary. Statistical model results

1. Surgery time

I. Model

The effect of explanatory variables on surgery time is estimated in a linear mixed model. The response is the log-transform of surgery time, the fixed variables are treatment (factor with two levels) and the covariates weight and age, the random structure is a random intercept and treatment effect by center as a random factor.

The model is run in R and lme4 with the following syntax.

```
lmer(log(surgery_time) ~ treat + weight + age + (1 + treat | centre), dat)
```

II. Anova results

Term	F	DF num	DF denom	p-value
Treatment	1.96	1	3.00	0.26
Weight	0.58	1	49.72	0.45
Age	2.39	1	60.46	0.13

III. Estimated means and relative ratio

Estimated means are calculated from the model and back-transformed to the response scale.

Treatment	Reponse	SE	DF	Conf. int lower	Conf. int upper
Control	16.73	0.73	2.33	14.19	19.73
LigaTie	21.26	4.04	2.99	11.60	38.97

The treatment effect is estimated from the model and back-transformed. Due to the log-transform the resulting contrast is the ratio between control and treatment.

Contrast	Ratio	SE	DF	t	p-value
Control / LigaTie	0.79	0.13	3	-1.4	0.26

A 95 percent confidence interval for the ratio is given by (0.46, 1.36). Applying that interval at the level of the overall estimated mean from the model (18.9 minutes) gives a back-transformed interval from the control being 10.3 minutes faster to 6.7 minutes slower.

IV. Testing centre-specific treatment effects

A centre-specific treatment effect is testing by running a model with and a model without that effect. The two models are compared with a likelihood-ratio test. The null hypothesis of that test will be that the treatment effect is the same across all centres.

The following R syntax is used.

```
mod1 <- lmer(log(surgery_time) ~ treat + weight + age + (1 | centre), dat)
mod2 <- lmer(log(surgery_time) ~ treat + weight + age + (1 + treat| centre), dat)
anova(mod1, mod2)
```

The result is given in the following table.

Test	Chisq	DF	p-value
Centre-specific treatment effect	32.0101	2	< 0.0001

2. Ligation time

I. Model

The effect of explanatory variables on ligation time is estimated in a linear mixed model. The response is the log-transform of ligation time, the fixed variables are treatment (factor with two levels) and the covariates weight and age, the random structure is a random intercept and treatment effect by center as a random factor.

The model is run in R and lme4 with the following syntax.

```
lmer(log(ligation_time) ~ treat + weight + age + (1 + treat | centre), dat)
```

II. Anova results

Term	F	DF num	DF denom	p-value
Treatment	0.00	1	3.00	0.96
Weight	19.34	1	60.67	< 0.01
Age	2.35	1	62.81	0.13

III. Estimated means and relative ratio

Estimated means are calculated from the model and back-transformed to the response scale.

Treatment	Reponse	SE	DF	Conf. int lower	Conf. int upper
Control	4.49	0.41	2.83	3.31	6.08
LigaTie	4.43	1.32	2.99	1.72	11.43

The treatment effect is estimated from the model and back-transformed. Due to the log-transform the resulting contrast is the ratio between control and treatment.

Contrast	Ratio	SE	DF	t	p-value
Control / LigaTie	1.01	0.23	3	0.06	0.96

A 95 percent confidence interval for the ratio is given by (0.49, 2.07). Applying that interval at the level of the overall estimated mean from the model (4.5 minutes) gives a back-transformed interval from the control being 2.3 minutes faster to 4.8 minutes slower.

IV. Testing centre-specific treatment effects

A centre-specific treatment effect is testing by running a model with and a model without that effect. The two models are compared with a likelihood-ratio test. The null hypothesis of that test will be that the treatment effect is the same across all centres.

The following R syntax is used.

```
mod1 <- lmer(log(ligation_time) ~ treat + weight + age + (1 | centre), dat)
mod2 <- lmer(log(ligation_time) ~ treat + weight + age + (1 + treat| centre), dat)
anova(mod1, mod2)
```

The result is given in the following table.

Test	Chisq	DF	p-value
Centre-specific treatment effect	32.0101	2	< 0.0001
