# Supplementary material for: Ligation of the canine mesovarium with a resorbable self-locking device: a multicentre study
Source: Acta Vet Scand. 2026 Sep 19;68:41. doi: 10.1186/s13028-026-00884-3 (PMC13613791; doi:10.1186/s13028-026-00884-3)
Supplement: Supplementary file 2 [file 13028_2026_884_MOESM2_ESM.pdf]

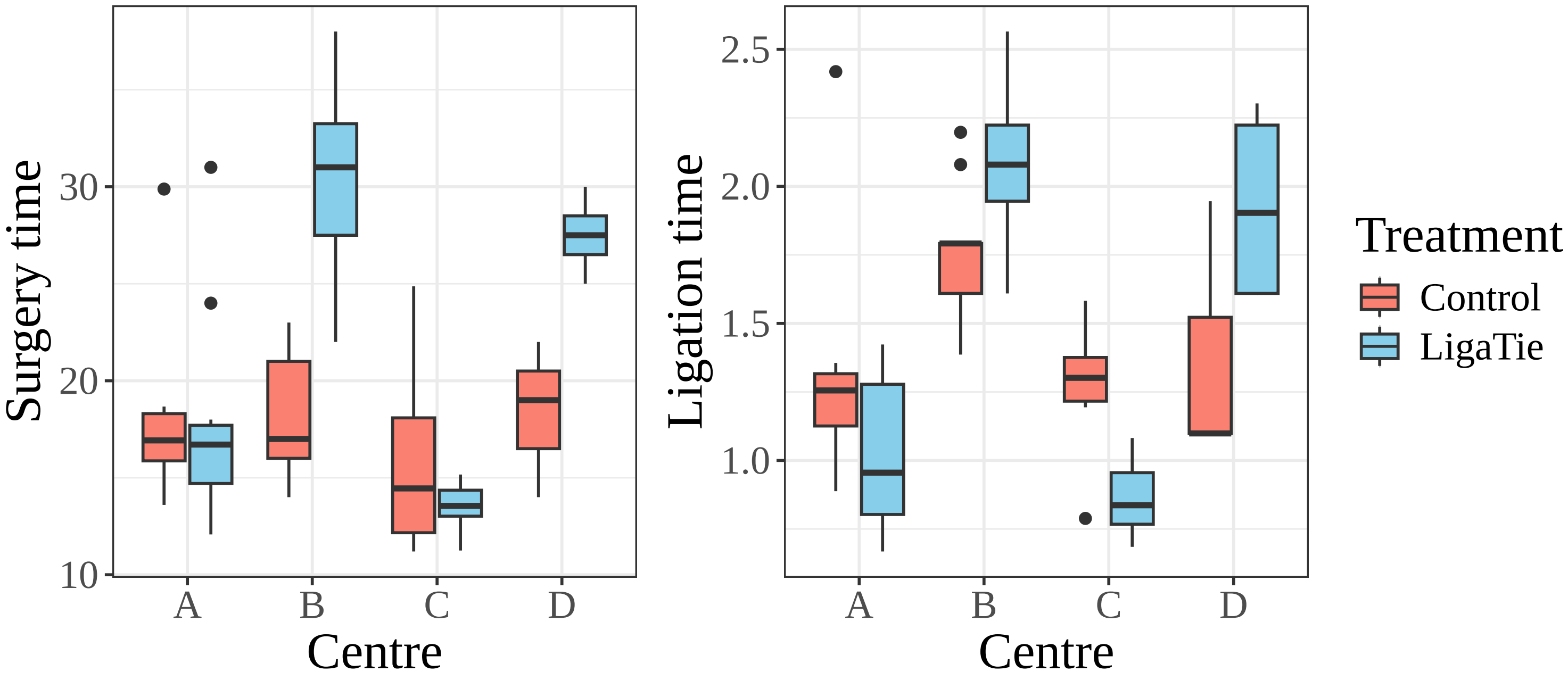

Surgery time
30
20
10
A
B
C
D
Centre
Ligation time
2.5
2.0
1.5
1.0
A
B
C
D
Centre
Treatment
Control
LigaTie
